# Supplementary material for: “It Feels Like My Spine is About to Break”: Experience and support needs of family caregivers of children with cerebral palsy in Ethiopia
Source: PLoS One. 2026 Apr 13;21(4):e0346049. doi: 10.1371/journal.pone.0346049 (PMC13075699; doi:10.1371/journal.pone.0346049)
Supplement: S1 File — (DOCX) [file pone.0346049.s001.docx]

**Appendix 1: Interview guide: caregiving experience of family caregivers of children with cerebral palsy in Ethiopia**

1. Could you please tell me about yourself and your child with CP?

- What is your relationship with the child?
- What do you know about your child’s condition (CP)? Probe: the cause?
- How long have you been caring for your child with CP?

1. How is your experience of caring for your child with CP?

- How is it to take care of ‘name of the child’?
- What kind of daily caregiving tasks do you carry out for your child with CP?
- What difficulties have you encountered?
- What helped you to overcome these difficulties?
- How have you coped with all the demands of caregiving?
- How have your challenges and coping strategies changed over time?
- Could you please share any positive experiences you gain from caregiving?

1. Could you tell me how caregiving has affected your own life?

- How has caring for a child with CP impacted your daily life and routines?
- How did it affect your family functioning (your relationship with your other children/your marriage/extended families/social life)?

1. How has caregiving affected your health?

- How has it affected your physical health (strain, back pain, fatigue)?
- How has it affected your mental health (probe: feeling of anxiety, stress, depression, feeling of guilt)

1. Could you please share with me any support needs you are experiencing?

- What are your pressing support needs in the caregiving course?
- What did you do to meet your needs?

1. What formal support services or helpful organizations are available to you?

- What kind of support did you get so far, and from whom??
- Is the support compatible with your needs?
- What kind of formal support do you wish you had?
- Do you have any suggestions for the improvement of existing support services?

1. Could you tell me your experience in obtaining support from CBR?

- How is CBR program supporting you and your child?
- Is the support compatible with your needs?
- What suggestion would you have for improvement of CBR support services for caregivers and children with CP?

1. What kind of natural support do you have?

- How has your family and community members supported you in caring for the child or through tough times?
- What kind of natural support do you wish you had?

1. What are your future concerns and aspirations for yourself and your child?

- What are your hopes and concerns for the future, both for yourself and your child with CP?
- What aspirations or goals do you have for your child with CP?

If you are able to take action to better support caregivers of children with cerebral palsy in Ethiopia, what would you do?

Probe: Looking back at your own experience, what do you wish for other caregivers to have? What would you change?

1. Do you have anything that you would like to raise and discuss?

**Thank you for your time and participation.**
