# Supplementary material for: “It Feels Like My Spine is About to Break”: Experience and support needs of family caregivers of children with cerebral palsy in Ethiopia
Source: PLoS One. 2026 Apr 13;21(4):e0346049. doi: 10.1371/journal.pone.0346049 (PMC13075699; doi:10.1371/journal.pone.0346049)
Supplement: Attachment — Submitted filename: Response letter-PLOSONE-reviewers.docx [file pone.0346049.s004.docx]

Date: 2025/08/22

To: Editor, PLOS ONE

**Response to reviewers’ comments**

We would like to thank you and the reviewers for the valuable feedback on ways to improve and strengthen our manuscript, titled: **“**It Feels Like My Spine is About to Break”: Experience and Support Needs of Family Caregivers of Children with Cerebral Palsy in Ethiopia. Below, we respond to each point raised by the reviewers, indicating any changes and their location in the manuscript with track changes. We have also attached a clean manuscript without track changes. Thank you for your consideration. We look forward to your response.

| **Reviewer’s comments** | | |
| --- | --- | --- |
| **Feedback** | **Authors response** | **Page/line #** |
| **Reviewer One** | | |
| 1. Literature review and Framing:   “It would be helpful to distinguish more clearly between findings from HICs and LMICs, both in the introduction and the discussion sections. | Thank you for your valuable feedback. We have revised the introduction as you suggested and presented the findings for HICs and LMICs separately. | Page 4-5/line 77-99 |
| The rationale for the importance of conducting the study in Ethiopia could be expanded and would help justify the study more fully. | Comment accepted and we revised the rationale to as follows: “A recent scoping review on the health outcomes and support needs of caregivers of children with CP in SSA highlighted a lack of research from Ethiopia, despite a growing body of evidence in the region (Melak et al., 2025). While previous studies from other countries could provide some insight, the unique socio-cultural and economic factors, such as the spiritual and collectivist nature of Ethiopian society (Karbo, 2013), multidimensional poverty (UNDP., 2022a), and stigma (Zuurmond et al, 2022) might influence the caregiving experience differently. Moreover, the limited availability of disability services and formal caregiving support systems in Ethiopia could impact caregiving for children with CP. To address this knowledge gap, the current study explored the caregiving experiences and support needs of family caregivers for children with CP in Ethiopia. Such a study is needed to provide a foundational evidence base to inform future research, as well as give insight into the design of relevant policies and support interventions tailored to the specific needs and realities of caregivers of children with CP in Ethiopia.” | Page 5-6/line 105-118 |
| Please clarify whether institutionalization or abandonment of children with CP is prevalent in the Ethiopian context. Since this option was mentioned by some mothers during the interviews, further contextualization would help the reader understand whether this reflects a broader social reality or isolated perceptions. | We appreciate your concern. We want to clarify that while some of our participants noted that they were initially considering the option of placing their child for adoption upon learning about their child's condition for the first time, they explained that this their initial reaction, largely due to a lack of information and support. However, none of the participants ultimately pursued this option; instead, they came to view their child's condition as a gift from God over time. Furthermore, we found no evidence of abandonment among children with CP within the Ethiopian context. | Page 17/line 309-312 |
| 2. Method:  The methodology section is well written, but the introduction could briefly preview the sampling rationale and characteristics (e.g., severe CP, broad age range). | Comment accepted, and we edited accordingly:  “Evidence showed that the most significant predictors of caregiver burden were the duration of caregiving and the level of dependency of the care recipient (Lindt et al.). Caregivers of children with CP were found to spend, on average, 15 hours per day in Turkey (Park & Nam, 2019) and 21.3 hours per day in Ethiopia (Kassa et al., 2024) on caregiving activities. Furthermore, mothers of children with CP with higher scores on the GMFCS tend to spend more time on caregiving than mothers of children with lower GMFCS scores (Ahmadi Kahjoogh et al., 2016). They also experience higher levels of stress due to increased caregiving demands, health concerns, and potential developmental delays. Similarly, as the child with CP gets older and heavier, the physical demand placed on caregivers also increases (Dambi et al., 2015). ” | Page 3/ line 63-64  & page 4/ line 67-71 |
| Further details on the recruitment process (e.g., how caregivers were contacted) would enhance transparency. | Based on your suggestion, we added the following: “Caregivers who were recruited for the study were contacted by CBR field workers to inquire about their willingness to participate in the research.” | Page 8/ line 183-185 |
| Are any of the children dependent on medical equipment (e.g., respiratory devices)? | No, none of the caregivers had a child with CP who uses medical equipment such as a respiratory device. |  |
| The setting description could be streamlined for focus and clarity. | We appreciate the feedback. We kept it as it was, as we think that offering a detailed description of the study setting is important to help readers better contextualize the findings and assess their potential transferability to similar settings. |  |
| Please provide more context for the use of the Multidimensional Model of Caregiving Process and Outcome, which informs the interview guide. | We accepted the comment, and we have edited as follows to provide more context:  “The interview guide was developed based on the Multidimensional Model of Caregiving Process and Outcome, developed by Raina et al. (2004), which provides a comprehensive understanding of the complex interplay between various factors that influence the caregiving experience. This model highlights how background/contextual factors (e.g., socioeconomic status), caregiver strain, intrapsychic factors and coping factors interact to affect caregiver outcomes such as physical and psychological health. The model informed the development of the interview guide, helping us identify key areas to explore, including caregivers’ day-to-day experiences, challenges, coping strategies, available support systems, and the broader contextual influences on caregiving” | Page 13/ line 206-213 |
| Consider explaining the predominance of girls with CP in the sample, in contrast with general statistics. | Thank you for your suggestion. We added this under the limitations of the study:  “Globally, CP is more prevalent among boys than girls (Ahmed et al., 2023; Chounti et al., 2013; Yang et al., 2021; Yeargin-Allsopp et al., 2008); however, the majority of caregivers in our sample were mothers of girls with CP. Future research should consider a gender-balanced sample and incorporate insights from fathers, siblings, grandparents, and caregivers from rural areas and diverse regions in Ethiopia to gain a comprehensive understanding of family caregiving for children with CP”. | Page 41/ line 872-873 &  Page 42/ line 877-879 |
| 3. Findings: Where possible, quantify or summarize the prevalence of key themes (e.g., “most parents reported…”), which can help convey the relative weight of each theme and guide the reader through the findings. | Thank you for your feedback. While we acknowledge the value of knowing the prevalence, we want to clarify that for this study, we followed the specific approach of reflexive thematic analysis (RTA) (Braun & Clarke, 2006, 2019, 2020). To make our work congruent with this approach, we generated rich, nuanced accounts of meaning rather than measured or quantified theme frequency. RTA views themes as patterns of shared meaning, rather than categories to be counted (Braun & Clarke, 2006, 2019, 2020). |  |
| In the section on acceptance and spirituality, some subthemes could be grouped under coping. Consider avoiding repetition of quotes or phrasing (e.g., regarding emotional growth or stigma). | We greatly appreciate your feedback and noticing some repetition. We have now integrated/moved the acceptance subtheme into the natural support subtheme, specifically integrating with the findings of spirituality as coping, as caregivers cited religion and spirituality as their reasons for accepting their child's condition. | Page 31/ line 617-635 |
| When referring to “natural support,” acknowledge that the same support sources may also cause distress, as was reflected earlier in the findings. | We appreciate the feedback. We edited accordingly:  **From:** “Caregivers received physical, informational, and emotional support from their natural support systems, such as family, friends, and neighbours.”  **To**: “Caregivers received physical, informational, and emotional support from their natural support systems, such as family, friends, and neighbours. However, the same support sources were reported as a source of distress in the earlier findings.” | Page 29/ line 573-575 |
| 4. Discussion:  The discussion would benefit from clearer articulation of how formal and natural support systems can be balanced. | We appreciate the suggestion. We have revised the discussion section:  **From:** “The findings of the current study imply that a balanced formal and natural support could enhance the positive experience of caregivers of children with CP.”  **To:** “The findings of the current study suggest that a balanced integration of formal and natural support could enhance caregivers' positive experiences. Therefore, it is crucial to improve the accessibility and availability of CBR support services. Recognizing the role of natural support systems and the roles of religion and spirituality in addressing the psychological and emotional needs of caregivers could have a sustainable impact on the well-being of caregivers. Therefore, fostering a system of collaboration between health professionals and religious leaders is vital.” | Page 39/ line 814-820 |
| The finding that most children were not enrolled in any educational framework deserves further attention. Is this reflective of systemic issues or specific to the sample? | Comment accepted and revision has been made accordingly. We have added to the discussion section as follows: “Moreover, Ethiopia’s commitment to inclusive education is affirmed in its ratification of CRPD. In contrast to this, in the current study, among the 13 children with CP, only one child was enrolled in school. This reflects the systemic issue rooted in structural, economic, and attitudinal barriers to inclusive education (Franck & Joshi, 2017; Tekola et al., 2020), where only 11% of children with special needs are enrolled in primary education and 2.8% in secondary education in Ethiopia (ESDP, 2021). Research evidence also shows that there is limited acceptance and retention of children with developmental disabilities in Ethiopia’s mainstream schools as compared to other disabilities due to their requirement of additional support (Okyere et al.; Tilahun et al., 2016). This evidence implies that the underdeveloped social services and systemic challenges impose an additional burden on caregivers. Therefore, the evidence highlights the urgent need for the full implementation of CRPD to enhance access to assistive technology, improve the accessibility of physical environments, and promote inclusive education, ultimately reducing the physical strain on caregivers.” | Page 38/ line 779-791 |
| I recommend that the gendered dimension of caregiving be highlighted more strongly. Since almost all caregivers were mothers**,** this calls for greater attention to gender-based inequities in care work, and the specific burdens mothers face in resource-limited settings. | Recommendation accepted, and we have added to the discussion section as follows: “Globally, deep-rooted gender norms result in family caregiving still being widely perceived as women’s work (Stall et al., 2023). As a result, women currently perform up to 81% of unpaid caregiver roles worldwide (Bhan et al., 2020). In the current study, all caregivers were women who assumed the responsibility of meeting their children's daily needs, managing household chores, and sometimes serving as the primary breadwinners. This finding highlights that caregiving responsibilities disproportionately fall on women, emphasizing that future interventions should consider the gendered inequities involved in caring for children with CP. Contrary to our finding, a recent study conducted in Saudi Arabia found that caregiving for children with CP was shared between fathers and mothers, indicating a shift from the traditional view of women as the primary caregivers (Alqahtani et al., 2025). ” | Page 37/ line 755-763 |
| Clarifying the practical relevance of the findings and their significance in the context of global caregiving challenges would help international readers better appreciate the contribution of the study. | Thank you for the feedback. We added the following to the conclusion:  “The findings offer insights into caregiving challenges shared across many low- and middle-income countries. Common challenges such as limited formal support, reliance on natural support systems, unmet needs and negative impact on the well-being of caregivers reflect broader global experiences. The influence of cultural and spiritual beliefs underscored the importance of contextually sensitive interventions. These insights contribute to a global understanding of caregiving and can inform the development of context-responsive policies and support practices.” | Page 42/ line 893-898 |
| Please note that functional classifications such as GMFCS and MACS were determined by parents rather than clinicians, which may limit the precision of these measures. | Although GMFCS, MACS, and CFCS classifications were based on caregivers’ perceptions, we minimized potential misclassification by providing clear guidance on the meaning of each level.  **We have modified from:** GMFCS, MACS, and CFCS tools were used to classify the functional ability of children with CP as perceived by caregivers.  **To this:** GMFCS, MACS, and CFCS tools were used to classify the functional abilities of children with CP based on caregivers’ perceptions, with guidance provided on the meaning of each level. | Page 9/ line 202-203 |
| **Reviewer 2** | | |
| Materials and Methods  1. There is no justification provided for the number of study participants (N = 13). The concern is not about the relatively small sample size itself – qualitative methodology does not require large samples – but rather about whether data saturation was achieved. What determined the decision to stop including additional caregivers in the study? According to qualitative methodological standards, this decision should be based on theoretical saturation of the collected material – that is, the point at which no new categories emerge from additional data. Was this the case in the present study? It remains unclear whether and how saturation was reached. The mention of "information power" is vague and does not clarify this point. | We greatly appreciate the concern. To clarify, consistent with our approach (exploratory qualitative descriptive), we were guided by the model of **information power** (the more relevant information a sample holds, the fewer participants are needed**)** (Malterud et al., 2015) to determine the adequacy of sample size. This model suggests that the size of a sample with sufficient information power depends on the study's aim, sample specificity, use of established theory, quality of dialogue, and analysis strategy**.**  Our study had a focused aim, the sample was specific, the interview guide was informed by the Multidimensional Model of Caregiving Process and Outcome, and interviews were conducted in-depth by an experienced interviewer in the participants’ native language, Amharic. These characteristics align with the principles of information power, making a sample size of 13 sufficient to address the research question. |  |
| 2. My next concern relates to the lack of anonymity in the study. The participants may not have perceived their participation as anonymous or confidential, given that they were required to sign written consent forms containing identifying information. This procedure may have reduced the participants’ openness during the interview and weakened the level of trust toward the researcher. Although the consent forms were later anonymized and the participants were assigned numerical identifiers, the act of signing one’s name is not a neutral action – especially when the interview involves emotional, intimate, and difficult topics. For this type of research, verbal consent – recorded together with the interview – would have been more appropriate. | We appreciate this feedback and acknowledge the importance of continuously reflecting on improving consent. We want to clarify that we followed the two ethical review board guidelines and their recommendations as to ethical research contact (as approved). We have added this to the limitation of the study: “Furthermore, given the sensitive nature of the topic, which encompassed emotional and intimate conversations, future researchers might consider obtaining verbal consent to enhance the openness and comfort of participants.” | Page 43/line 881-883 |
| 3. The research tool attached (Appendix 1) includes only broadly formulated questions. There is no indication of any follow-up or probing questions the researcher used to explore specific topics in depth. | We have added the follow-up questions used in the interview guide. |  |
| Findings:  4. I have reservations about the way the research findings are presented. Here we have an abundance of quotes and very modest, shallow interpretations by the authors (especially in Topic 1). Although the use of the participants’ direct statements strengthens credibility, deepens the analysis, and superbly illustrates the emotions and context, the authors’ commentary is too sparse and lacks analytical depth. Description clearly dominates here, without deeper conceptualizations. The interpretative potential of the collected qualitative material should be utilized more thoroughly. | We appreciate the concern. However, we would like to clarify that the study was exploratory descriptive (Hunter, 2019). Following Hunter, (2019) approach, we adhered closely to the participants' words, as recommended. We acknowledge that using an alternative approach could yield a different presentation of the findings; however, this would constitute a different study. |  |
| 5. The presented research findings also lack individual differences, descriptions of various cases beyond the main themes, which contradicts the standards for reporting qualitative data (e.g., COREQ checklist, 2007, item 32). The caregivers’ experiences may vary depending on the sociodemographic characteristics, family situation, or attitude toward religion. While reading the findings, I wondered about the answers to the following questions:  -Does the level of caregivers’ education influence their understanding of the diagnosis?  -Does the family situation (e.g. divorce) affect the adaptation process to the child’s disability?  -Does having more children influence the process of coming/the ability to come to terms with the child’s illness?  -Was faith in God a facilitating factor in coping with the child’s disability for every caregiver?  -Was this true for every caregiver who declared themselves as Orthodox Christians?  -In the context of care for a child with CP,  does the birth order of the affected child matter?  -Did the ongoing civil war in the study region (Amhara) impact the participants’ experiences (for example, through worsening of their economic situation and causing additional stress)?  I am convinced that data on secondary themes/experiences of the caregivers could make the analyses more diverse and even more interesting. Highlighting individual differences could successfully broaden and deepen the authors’ interpretations, as qualitative research is not about just citing respondent statements, which in the text are numerous, lengthy, and therefore overrepresented. | Thank you for the feedback. Given that all participants were recruited from a single community-based rehabilitation (CBR) program in Gondar city, we think that providing further individual-level sociodemographic details particularly in combination with direct quotes would risk breaching participant confidentiality, as individuals could potentially be identified. Protecting participant privacy was a key ethical consideration, especially in a relatively small and specific sample (Kaiser, 2009).  Moreover, the aim of this study was to explore the **common caregiving experiences and support needs** of family caregivers of children with CP, rather than to analyze variation across sociodemographic groups because we didn’t consider maximum variation of the samples beyond the severity of CP and the age of the child. Our analytical approach followed a **cross-case analysis**, consistent with the **exploratory qualitative descriptive design**, and was focused on identifying shared patterns across participants, rather than conducting comparisons based on individual characteristics. Still, we believe that this will be important to be addressed in future studies.  -We have included the following statement about the impact of regional conflict, which exacerbated the financial difficulties of caregivers:  The ongoing conflict and instability in the region have exacerbated their economic challenges, along with the rising cost of living. One caregiver explained: “Due to the war, I lost my source of income. At times, we didn’t even have anything to eat. I started a small business on the street to meet some of my children’s needs, but as you can see, the situation [the inflation and instability] in the country has worsened. It’s hard to earn any income from that now.” (P-7).  - We already included **positive caregiving outcomes** (e.g., personal growth, resilience) as a **sub-theme, contrasting with the main theme, which mainly indicated the burden of caregiving on physical health and mental and social well-being,** to illustrate the complexity of caregiving experiences. | Page 34/ line 696-700  Page 24/ line 536-570 |
| 6. The analysis of the empirical material could be deepened by incorporating field notes, which the authors only briefly mention. From the text, we know that the researcher made them after the interviews, but we do not know whether their content was consistent with the interviews. I am curious if they included the researcher’s observations regarding the caregivers’ health functioning (visible spinal problems, possible difficulties with walking, or overall physical fitness). How does the lack of assistive equipment (e.g. standing frames, hoists) affect the caregivers’ physical health? It is impossible to conduct an interview without also performing participant observation. | We want to clarify that structured participant observation was not part of this study. However, we incorporated field notes to contextualize the interview data and to support the reflexive thematic analysis. The data collector documented her observations such as the caregivers’ appearance, emotional expressions during data collection, and her reflections immediately following each interview. These notes were referred to during data familiarization and theme development when needed.  We have revised the manuscript to clarify the role of field notes in the analysis process.  We revised as follows:  **Under data collection:** During and immediately after each interview, the interviewer wrote field notes to capture non-verbal cues and reflections.  **Under data analysis:** First, to gain a better contextual understanding of the data, MM familiarized herself with the data by repeatedly listening to the audio-recorded data, reading the Amharic transcripts, and reviewing the field notes. | Page 10/ 219-220  Page 10/ 228-230 |
| Recommendations for Development of the Research Project:  1. Due to the highly diverse social structure of Ethiopia, in my opinion, the research should be continued and extended to rural populations or other regions of the country. The recruitment was limited to participants from a city covered by a single CBR program (Gondar), which weakens the transferability of the results. 2. Although mothers are the main caregivers, their exclusive participation may limit insight into family care processes. The study could benefit from including fathers, grandparents, or siblings of children with CP. | Thank you for your suggestion, and we added these points in the limitations and future research direction section. | Page 42/ 871-879 |

**References**

Ahmadi Kahjoogh, M., Rassafiani, M., Tahmasebi, A., Ahmadi Kahjoogh, H., & Sahaf, R. (2016). The relationship between gross motor function ability and time use in mothers of children with cerebral palsy. *British Journal of Occupational Therapy*, *79*(3), 172-177. <https://doi.org/10.1177/0308022615616974>

Ahmed, A., Rosella, L. C., Oskoui, M., Watson, T., & Yang, S. (2023). Prevalence and temporal trends of cerebral palsy in children born from 2002 to 2017 in Ontario, Canada: Population-based cohort study. *Dev Med Child Neurol*, *65*(2), 243-253. <https://doi.org/10.1111/dmcn.15324>

Alqahtani, A., Sahely, A., Aldersey, H. M., Finlayson, M., Macdonald, D., & Fakolade, A. (2025). Understanding the Parental Caregiving of Children with Cerebral Palsy in Saudi Arabia: Discovering the Untold Story. *Int J Environ Res Public Health*, *22*(6). <https://doi.org/10.3390/ijerph22060946>

Bhan, N., Rao, N., & Raj, A. (2020). Gender Differences in the Associations Between Informal Caregiving and Wellbeing in Low- and Middle-Income Countries. *Journal of Women's Health*, *29*(10), 1328-1338. <https://doi.org/https://doi.org/10.1089/jwh.2019.7769>

Chounti, A., Hägglund, G., Wagner, P., & Westbom, L. (2013). Sex differences in cerebral palsy incidence and functional ability: a total population study. *Acta Paediatr*, *102*(7), 712-717. <https://doi.org/10.1111/apa.12240>

Dambi, J. M., Jelsma, J., & Mlambo, T. (2015). Caring for a child with Cerebral Palsy: The experience of Zimbabwean mothers. *Afr J Disabil*, *4*(1), 168. <https://doi.org/10.4102/ajod.v4i1.168>

Franck, B., & Joshi, K. (2017). Including students with disabilities in education for all: Lessons from Ethiopia. *International Journal of Inclusive Education*, *21*(4), 347-360.

Karbo, T. (2013). Religion and social cohesion in Ethiopia. *International Journal of Peace and Development Studies*, *4*(3), 43-52.

Kassa, T., Tadese, H., Eriku, G. A., Abich, Y., & Fentanew, M. (2024). Health-related quality of life and associated factors among primary caregivers of children with cerebral palsy, in Bahir Dar and Gondar cities, Ethiopia, 2022. *PLoS One*, *19*(4), e0301050.

Lindt, N., van Berkel, J., & Mulder, B. C. Determinants of overburdening among informal carers: a systematic review.

Melak, M., Fakolade, A., Mekonnen, S., Baraki, A., Ross-White, A., & Batorowicz, B. (2025). The state of evidence on the health outcomes and support needs of family caregivers of children with Cerebral Palsy in Sub-Saharan Africa: a scoping review. *Disability and rehabilitation*, 1-16. <https://doi.org/https://doi.org/10.1080/09638288.2025.2472984>

Okyere, C., Aldersey, H. M., Lysaght, R., & Sulaiman, S. K.

Park, E.-Y., & Nam, S.-J. (2019). Time burden of caring and depression among parents of individuals with cerebral palsy. *Disability and rehabilitation*, *41*(13), 1508-1513.

Stall, N., Shah, N., & Bhushan, D. (2023). Unpaid Family Caregiving—The Next Frontier of Gender Equity

in a Postpandemic Future. *JAMA Health Forum*. <https://doi.org/doi:10.1001/jamahealthforum.2023.1310>

Tekola, B., Kinfe, M., Girma, F., Hanlon, C., & Hoekstra, R. A. (2020). Perceptions and experiences of stigma among parents of children with developmental disorders in Ethiopia: A qualitative study. *Soc Sci Med*, *256*, 113034. <https://doi.org/10.1016/j.socscimed.2020.113034>

Tilahun, D., Hanlon, C., Fekadu, A., Tekola, B., Baheretibeb, Y., & Hoekstra, R. A. (2016). Stigma, explanatory models and unmet needs of caregivers of children with developmental disorders in a low-income African country: a cross-sectional facility-based survey. *BMC Health Serv Res*, *16*, 152. <https://doi.org/10.1186/s12913-016-1383-9>

UNDP. (2022a). *Ethiopia-Working Paper Series: Crisis, Resilience and Opportunity: Poverty, Human Development, and the Macro-Economy in Ethiopia, 2020-23* (Working Paper Series, Issue. <https://www.undp.org/ethiopia/publications/undp-ethiopia-working-paper-series-crisis-resilience-and-opportunity-poverty-human-development-and-macro-economy-ethiopia-2020>

Yang, S., Xia, J., Gao, J., & Wang, L. (2021). Increasing prevalence of cerebral palsy among children and adolescents in China 1988-2020: A systematic review and meta-analysis. *J Rehabil Med*, *53*(5), jrm00195. <https://doi.org/10.2340/16501977-2841>

Yeargin-Allsopp, M., Van Naarden Braun, K., Doernberg, N. S., Benedict, R. E., Kirby, R. S., & Durkin, M. S. (2008). Prevalence of cerebral palsy in 8-year-old children in three areas of the United States in 2002: a multisite collaboration. *Pediatrics*, *121*(3), 547-554. <https://doi.org/10.1542/peds.2007-1270>
