# Supplementary material for: “It Feels Like My Spine is About to Break”: Experience and support needs of family caregivers of children with cerebral palsy in Ethiopia
Source: PLoS One. 2026 Apr 13;21(4):e0346049. doi: 10.1371/journal.pone.0346049 (PMC13075699; doi:10.1371/journal.pone.0346049)
Supplement: Attachment — Submitted filename: Response to reviewers-Melkitu M.docx [file pone.0346049.s005.docx]

Date: 2025/11/16

To: Editor, PLOS ONE

**Response to reviewers’ comments**

We would like to thank you and the reviewers for the second round of valuable feedback to improve and strengthen our manuscript, entitled: **‘**It Feels Like My Spine is About to Break’: Experience and Support Needs of Family Caregivers of Children with Cerebral Palsy in Ethiopia. Below, we respond to each point raised by the reviewers, indicating any changes and their location in the manuscript with track changes. We have also attached a manuscript without track changes. Thank you for your consideration. We look forward to your response.

| **Reviewer one** | **Authors response** | **Page#/Line # (manuscript with track changes)** |
| --- | --- | --- |
| 1. Authers stated that "While previous studies from other countries could provide some insight, the unique socio-cultural and economic factors, such as the spiritual and collectivist nature of Ethiopian society (Karbo, 2013), multidimensional poverty (UNDP., 2022a), and stigma (Zuurmond et al, 2022) might influence the caregiving experience differently"- the term “stigma” remains too general and vague. It would be helpful to clarify what kind of stigma is referred to, and to whom. | We have revised and changed stigma” to  “Community members' negative attitudes towards children with disability .” | Page 5/ line 96 |
| 2. Disability services and formal caregiving support – The mention of the limited availability of services and support systems in Ethiopia is important, but somewhat underdeveloped. I encourage the authors to either provide supporting references or expand on concrete details regarding the types of services lacking and how this impacts caregiving for children with CP. | We have added details about the types of services with supporting references as below:  Moreover, in Ethiopia, there is limited availability and accessibility of disability support systems, including community-based and specialized rehabilitation services and healthcare services (Asres; Atnafu et al., 2025), educational services for children with disability (Taddese et al., 2025; Tekola et al., 2023), caregiver benefits (Tekola et al., 2023) , and day care and respite care services (Aldersey et al., 2020; Jansen-van Vuuren et al.). These challenges were linked by researchers to increased physical, financial, and emotional demands of caregiving and were found to negatively impact the overall well-being of caregivers (Kassa et al., 2024; Szlamka et al., 2023). | Page 5/ line 97-104 |
| 3. Impact of conflict and instability – The authors added valuable examples of how the ongoing conflict and instability in the region affect economic challenges. However, I wondered whether there are more specific examples relevant to caregiving itself (e.g., access to rehabilitation, transportation difficulties, or disruptions to medical or educational services). | To further clarify, aside from economic challenges, which we reported, our study participants did not mention or discuss any additional conflict-related content. Thus, we included all the findings from our study in relation to conflict. |  |
| 4. Moreover, if the authors address the critical nuance of caregiving in a conflict zone (which I think adds complexity and relevance), I recommend expanding the background and/or discussion to include theoretical or empirical literature on the impact of protracted political conflict or war on quality of life and caregiving for children with disabilities, including CP. This would add depth and situate the findings within a broader scholarly framework. | To follow your suggestion, which we appreciated, we have added a paragraph in the discussion placing our findings within the body of existing evidence on conflict. Specifically, we added:  Our study found that the civil war in the region exacerbated caregivers' economic difficulties. Other researchers reported similar findings highlighting how living in the areas of protracted political conflict increases the inequalities and vulnerabilities of children with disabilities and their caregivers, and poses challenges that result in economic loss and diminished well-being (Karni-Visel Y et al., 2023). A study from Israel showed that war negatively impacted not only the economic status but also psychosocial health of caregivers of children with developmental disabilities. The authors found that during wartime, there were disruptions of educational and rehabilitation services, leading to missed work, unexpected treatment costs, and increased caregiving demands, particularly impacting parents with low economic status (Karni-Visel Y et al., 2023; Karni-Visel et al., 2025). Similarly, families and children with disabilities affected by armed conflict in Syria were reported to face significant challenges, such as losing their homes, livelihoods, and essential resources. These families struggled to meet basic needs, including food, healthcare, housing, assistive devices, medication, therapies, and transportation to access necessary services (Ćerimović, 2023). Overall, the findings of our and other studies These findings indicate the need to safeguard essential disability-related services during periods of conflict and to provide targeted financial support to economically vulnerable caregivers. | Page 40/from line 839-852 |
| **Reviewer 2** | **Authors response** | **Page/line #** |
| 1.No explicit statement that data saturation was achieved; instead, the author relies solely on the concept of “information power,” which does not fully meet the reviewer’s expectations, | We would like to further clarify that our approach is based on established methodological evidence and previously published **exploratory qualitative descriptive studies,** which indicated that information power, rather than data saturation, should guide sample size determination, particularly when reflexive thematic analysis is considered. Below, we provide the supporting evidence from key researchers in the field of thematic analysis methodology for our decision to choose information power over saturation:   - As per Braun and Clarke (2019), because saturation is embedded in the logic of Grounded Theory, it is not conceptually aligned with reflexive thematic analysis. Braun and Clarke (2019) instead recommend information power which was coined by Maltrud et al., 2016 as an appropriate framework for determining sample adequacy. - Information power, which shifts the focus from “Have I stopped hearing anything new?” to “Does my sample hold sufficient, relevant, and meaningful information to address the study aim?” aligns with an exploratory qualitative descriptive approach, where the goal is to provide varied and sufficiently rich accounts (Hunter David et al., 2019). - Recently published exploratory qualitative descriptive studies in health and rehabilitation sciences have adopted this approach to justify sample adequacy (Engeda et al.; Gaurav et al., 2023; Vuuren et al., 2023). | Page 8/ line 176-195 |
| 2.Limited response to concerns about “shallow interpretation”—rather than deepening the analysis, the author primarily defended the chosen methodological approach (exploratory-descriptive). | We want to further clarify that we closely followed the selected Exploratory Descriptive Qualitative design, and four authors engaged in the iterative process following specific steps of Reflexive Thematic Analysis as outlined by Braun and Clarke (2020), which led to our reported findings.  However, this reviewer’s comment prompted us to reflect more on the findings and subsequently we revised the discussion to enhance clarity of our interpretation of the findings; we included additional research evidence to situate findings with the broader global evidence. | Page 36-42 |
| 3.Adding a brief note stating that after a certain number of interviews no new categories emerged (which would suggest that saturation was indeed achieved), | Please refer our response above regarding data saturation (also please see our response to feedback 1). Consequently, we didn’t add the “no new category” statement. However, to enhance clarity, we revised the description in methods to:  Consistent with an exploratory, descriptive, qualitative approach, sample size adequacy was guided by the concept of information power which indicates the more information the sample holds, relevant for the study, the fewer participants is needed (Braun & Clarke, 2019; Malterud et al., 2016). The size of a sample with sufficient information power depends on five factors: (a) the aim of the study, (b) sample specificity, (c) use of established theory, (d) quality of dialogue, and (e) analysis strategy (Malterud et al., 2016). For the current study, the aim of this study was to gain understanding of the caregiving experience of family caregivers of children with CP in Ethiopia, sample included only primary family caregivers of children with severe CP, the Multidimensional Model of Caregiving Process and Outcome guided the study (Raina et al., 2004), and the method of analysis was a thematic cross-case approach (Malterud et al., 2015).To enhance the quality of dialogue during data collection, participants were selected purposively, considering their ability to articulate independent ideas in Amharic language. Therefore, the aim of the study was narrow, samples were specific, conceptual framework was used and had quality dialogue between the interviewer and caregivers which all indicated that a small sample size is needed for our study. We decided to stop at 13 participants, as the data we obtained were rich and sufficient to answer our research question. | Page 8/ line 176-195 |
| 4. Adding 2–3 additional sentences in the discussion to further elaborate on interpretation, for example by linking the findings to the broader literature to demonstrate that the analytical potential of the data was fully utilized within the chosen approach. | We have further revised the discussion by connecting our findings to broader global research evidence. (See also our response to feedback # 2) | Page 36/ line 737-747,  Page 37/ line 755-765,  772-775,  Page 37-39/line 783-789,  805-815,  Page 39/from line 819-831  Page 40/ line 834-852  Page 41-42/ line 865-868,  872-878,  879-883 |

**Thank you!!**

**References**

Aldersey, H. M., Ahmed, A. N., Tesfamichael, H. N., & Lotoski, N. (2020). Needs of families of children with intellectual and developmental disabilities in Addis Ababa. *Afr J Disabil*, *9*, 735. <https://doi.org/10.4102/ajod.v9i0.735>

Asres, G. W. The practices of community-based rehabilitation program for people with disabilities for inclusive development, Ethiopia.

Atnafu, D., Kuper, H., & Mbazzi, B. (2025). Left behind in primary healthcare: A qualitative exploration of healthcare experiences of people with disabilities in Ethiopia. *PLOS Global Public Health 5*(9). <https://doi.org/https://doi.org/10.1371/journal.pgph.0005147>

Braun, V., & Clarke, V. (2019). To saturate or not to saturate? Questioning data saturation as a useful concept for thematic analysis and sample-size rationales. *Qualitative Research in Sport, Exercise and Health*, *13*(2), 201–216. <https://doi.org/https://doi.org/10.1080/2159676X.2019.1704846>

Ćerimović, E. (2023). At risk and overlooked: Children with disabilities and armed conflict. *International Review of the Red Cross*, *105*(922), 192–216. <https://doi.org/https://doi.org/10.1017/S181638312200087X>

Engeda, E. H., Aldersey, H. M., Davison, C. M., Gelaye, K. A., & Fayed, N.

Gaurav, N., Aldersey, H., Lewis, J., & Batorowicz, B. (2023). Designing schools for all: Indian architects’ perspectives about physical disability and disability-related school design needs. *International Journal of Educational Development*, *100*. <https://doi.org/https://doi-org.proxy.queensu.ca/10.1016/j.ijedudev.2023.102786>

Hunter David, McCallum Jacqueline, & Howes Dora. (2019). Defining Exploratory-Descriptive Qualitative (EDQ) research and considering its application to healthcare. *Journal of Nursing and Health Care (JNHC)*, *4*.

Jansen-van Vuuren, J., Dawud, S., Lysaght, R., Batorowicz, B., & Aldersey, H. M. Family quality of life and children with disability in Ethiopia: The role of support providers.

Karni-Visel Y, Roth D, Ali-Saleh Darawshy N, & Schertz M. (2023). Quality of life between the hammer and the anvil:Challenges of living with a disability in areasof protracted political conflict. *Journal of Policy and Practice in Intellectual Disabilities*. <https://doi.org/https://doi.org/10.1111/jppi.12472>

Karni-Visel, Y., Roth, D., Lev, S., & Werbeloff, N. (2025). Quality of life and mental health in families of children with developmental disabilities during wartime. *Psychological Trauma: Theory, Research, Practice, and Policy*, *17*(7), 1434–1442. <https://doi.org/https://doi.org/10.1037/tra0001941>

Kassa, T., Tadese, H., Eriku, G. A., Abich, Y., & Fentanew, M. (2024). Health-related quality of life and associated factors among primary caregivers of children with cerebral palsy, in Bahir Dar and Gondar cities, Ethiopia, 2022. *Plos one*, *19*(4), e0301050.

Malterud, K., Siersma, D., & Guassora, D. (2015). Sample size in qualitative interview studies: guided by information power. *Qualitative Health Research*, *2613*, 1753–1760.

Malterud, K., Siersma, V. D., & Guassora, A. D. (2016). Sample Size in Qualitative Interview Studies: Guided by Information Power. *Qual Health Res*, *26*(13), 1753–1760. <https://doi.org/10.1177/1049732315617444>

Raina, P., O'Donnell, M., Schwellnus, H., Rosenbaum, P., King, G., Brehaut, J., Russell, D., Swinton, M., King, S., Wong, M., Walter, D., & Wood, E. (2004). <caregivingburden and care givng stress CF to guide research.pdf>.

Szlamka, Z., Ahmed, I., Genovesi, E., & al., e. (2023). Conceptualising the empowerment of caregivers raising children with developmental disabilities in Ethiopia: a qualitative study. *BMC Health Serv Res*, *1420*. <https://doi.org/https://doi.org/10.1186/s12913-023-10428-4>

Taddese, T., Gebresilase, M., Aboudahr, M., Dinsa, M., & Aslam, S. (2025). The practice of inclusive education in Central Ethiopia Regional State: a case study of primary schools. *International Journal of Primary, Elementary and Early Years Education*, 1–14. <https://doi.org/https://doi.org/10.1080/03004279.2024.2449462>

Tekola, B., Kinfe, M., Girma Bayouh, F., Hanlon, C., & Hoekstra, R. A. (2023). The experiences of parents raising children with developmental disabilities in Ethiopia. *Autism*, *27*(2), 539–551. <https://doi.org/10.1177/13623613221105085>

Vuuren, J., Dawud, S., Lysaght, R., Batorowicz, B., & Aldersey, H. (2023). Family quality of life and children with disability in Ethiopia. *African Journal of Disability 12*(12(0), a1124). <https://doi.org/> <https://doi.org/10.4102/ajod.v12i0.1124>
