# Supplementary material for: “It Feels Like My Spine is About to Break”: Experience and support needs of family caregivers of children with cerebral palsy in Ethiopia
Source: PLoS One. 2026 Apr 13;21(4):e0346049. doi: 10.1371/journal.pone.0346049 (PMC13075699; doi:10.1371/journal.pone.0346049)
Supplement: Attachment — Submitted filename: Point by point response-3rd round.docx [file pone.0346049.s006.docx]

Date: 2026/02/28

To: Editor, PLOS ONE

**Response to reviewers’ comments**

We would like to thank you and the reviewer for the third round of valuable feedback to improve and strengthen our manuscript, entitled: **‘**It Feels Like My Spine is About to Break’: Experience and Support Needs of Family Caregivers of Children with Cerebral Palsy in Ethiopia. Below, we respond to each point raised by the reviewer one, indicating any changes and their location in the manuscript with track changes. We have also attached a manuscript without track changes. Thank you for your consideration. We look forward to your response.

| **Reviewer one** | **Authors response** | **Page#/Line # (manuscript with track changes)** |
| --- | --- | --- |
| 1. In the statement, “In this context, caregiving could impose a double burden on women who already have demanding day-to-day roles and might also exclude them from participation in all areas of life, such as education, employment, and decision-making, which in turn affect their ability to provide optimal care for their child with CP,” the authors are encouraged to clarify what is meant by “decision-making.” Specifically, it would be helpful to distinguish whether this refers to household decision-making, healthcare-related decisions, or broader social and community-level participation. In addition, the authors should more explicitly explain the mechanisms through which exclusion from decision-making may influence the quality of care provided to children with CP. | Thank you for the constructive feedback. We have clearly updated the statement as recommended. **From this:**  “In this context, caregiving could impose a double burden on women who already have demanding day-to-day responsibilities and might also exclude them from participation in all areas of life, such as education, employment, and decision-making, which in turn affects their ability to provide optimal care for their child with CP.”  **Into this:**  “In this context, caregiving could impose a double burden on women who already have demanding day-to-day responsibilities and might also exclude them from participation in education, paid employment, and political and social engagement (46, 71). Such exclusion could reduce their access to resources, autonomy in household and healthcare decision-making, and impact their well-being, ultimately affecting their ability to provide quality care for their child with CP.” | Page 35, line 726-730 |
| 2. The issue of providing accurate psychoeducational information and addressing misconceptions is highly important; however, this section would benefit from clearer articulation. Specifically, the authors are encouraged to clarify how the proposed strategies (e.g., multidisciplinary rehabilitation, professional guidance, and training religious leaders) contribute to improving caregivers’ understanding and satisfaction. In addition, it would strengthen the discussion to support these recommendations with references to empirical studies demonstrating that such approaches have been effective in similar contexts. | We have clearly articulated the statement and also provided supporting evidence.  **From this**: “Therefore, it is necessary to ensure access to comprehensive multidisciplinary rehabilitation therapy to maximize the functional outcomes for children with CP and enhance caregivers' satisfaction with services. In addition, health care professionals need to provide caregivers with accurate, clear information about CP. Training religious leaders could also facilitate the dissemination of information to caregivers (94).”  **Into this**: “……This reflects caregivers’ misconceptions that CP is a curable condition through spiritual practices. Consistent with our study findings, research from Ethiopia has shown that family caregivers of people with chronic health conditions often engage in religious rituals and services to seek miraculous healing and emotional comfort (95). Therefore, healthcare and rehabilitation professionals need to equip caregivers with clear and accessible health information about the cause and prognosis of CP through educational training programs. In a similar context, educational workshops by health care professionals for caregivers of children with CP have improved caregivers' knowledge and health outcomes for the children and their caregivers in Zimbabwe (96, 97). In addition, research has shown that multidisciplinary rehabilitation intervention improved functional motor skills and quality of life, as well as social, emotional, and cognitive outcomes for children with disabilities, and also enhanced satisfaction and helped family caregivers feel more supported and empowered in their care (96, 97). Therefore, it is necessary to ensure access to multidisciplinary rehabilitation services for children with CP. Beyond the health care professionals, training religious leaders and equipping them with accurate information about CP could help address misconceptions and guide caregivers toward appropriate services, as faith leaders are important and trusted members of the community (33, 98). The World Health Organization (WHO) also recognizes the crucial role of faith leaders in supporting the spread of reliable health information and promoting positive health behaviours, specifically in LMICs (99, 100).” | Page 40-41, line 856-877 |
| 3. A proofreading of the Discussion section is required, as it contains several language and phrasing issues. (e.g., the phrase “Contrary to study” is one example and should be revised to “Contrary to the present study” or “In contrast to the findings of this study”); punctuation should be added where necessary etc. | We have thoroughly read and corrected the phrasal and grammatical issues, as well as the punctuation, throughout the discussion section. |  |
